# Transmural invasion of hepatic flexure of colon causing cholecystocolic fistula by aggressive gallbladder carcinoma

**DOI:** 10.1186/1477-7819-11-86

**Published:** 2013-04-16

**Authors:** Amit Nandan Dhar Dwivedi, Satendra Kumar, Samir Rana, BabuNandan Maurya

**Affiliations:** 1 Department of Radiodiagnosis and Imaging, Institute of Medical Science, Banaras Hindu University, Varanasi, 221005, India; 2 Department of General Surgery, Institute of Medical Science, Banaras Hindu University, Varanasi, 221005, India

**Keywords:** Cholecystocolic fistula, Gallbladder carcinoma, Multidetector CT

## Abstract

Spontaneous enterobiliary fistulae are a complication of biliary disease or a disease of adjacent structures. Cholecystocolic fistulae are rare in relation to gallbladder carcinoma (GBC). Previous reports have presented images showing subtle findings suggestive of cholecystocolic fistula. We report the unusual spread and rare images of a case of cholecystocolicfistula,to highlight the aggressive nature of GBC and findings of gross transmural invasion of the colonic wall. The images acquired in all three planes define the anatomical and pathological extent conclusively. There are a higher number of GBC cases across the geographic belt of North India compared to the West. In this case, the patient’s pathology was extensive and unresectable, and therefore palliative and supportive care wasadvised.

## Background

The cholecystocolonic fistula is an uncommon but pertinent complication of gallbladder disease, occurring in 0.06 to 0.14% of patients with biliary disease [1,2]. Among the different types of cholecystoenteric fistulas, the cholecystoduodenal is the most common with cholecystocolonic fistulas being the second most common [3]. Aggressive gallbladder carcinomas (GBCs) rarely invade into the adjacent duodenum and/or colon resulting in internal biliary fistula. Worldwide epidemiological studies have implicated dietary factors in the development of GBC. The ecological evidence indicates considerable geographic variation in the incidence of GBC. Variations in the incidence of various populations might be partly determined by dietary variations. Patients may present with non-specific symptoms such as diarrhea, malena and loss of weight. Barium studies of the gastrointestinal tract and colon are diagnostic. Multidetector computed tomography (MDCT) can demonstrate the fistulous communication and anatomical details in all three planes.

## Case presentation

We discuss the case of a 48-year-old woman who presented with right hypochondrial pain, jaundice and melena. On examination there was severe jaundice and a lump in the right hypochondrium. The patient underwent an abdominal ultrasonography which showed gallbladder fossa mass infiltrating the portahepatis with proximal biliary dilatation. Fine needle aspiration cytology (FNAC) was undertaken and revealed high grade adenocarcinoma. The patient was advised MDCT evaluation. Contrast enhanced multiplanar CT (computed tomography; 64-slice LightSpeed GE scanner; GE Healthcare, Waukesha, WI, USA) with IV and oral contrast was performed.

**Figure 1:**
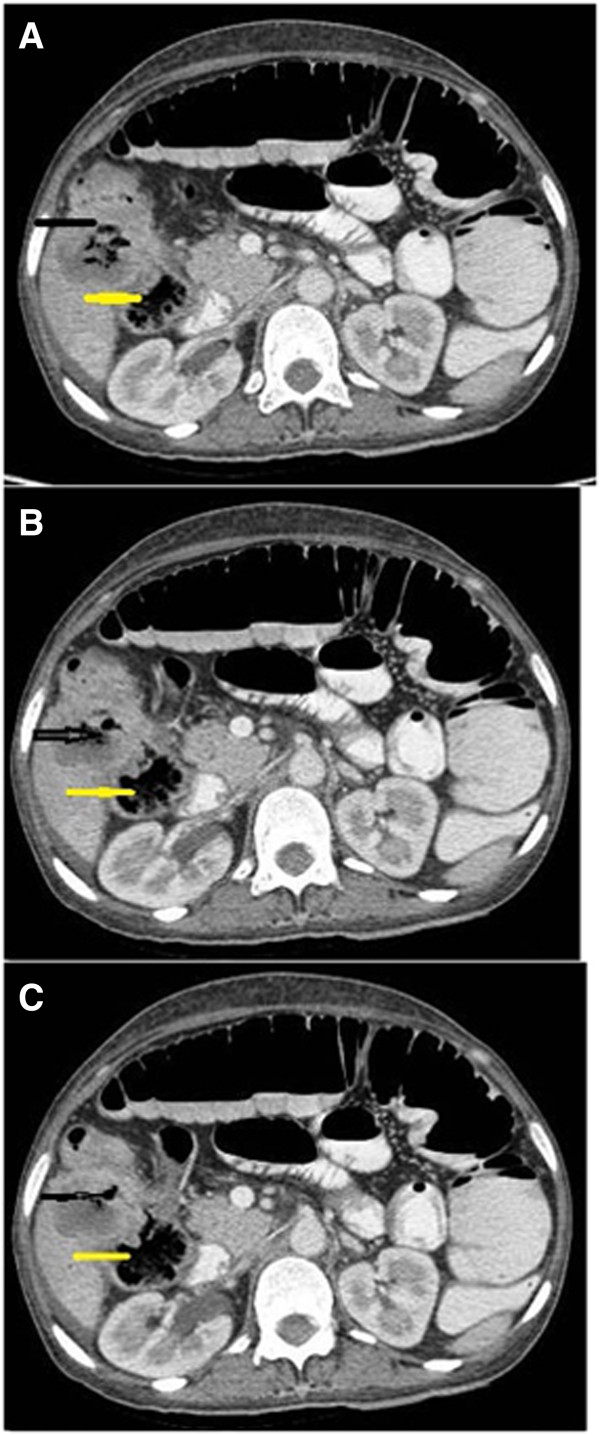
**A-C Multiple air loculi visible within the mass lesion (black arrow).** Contiguous axial sections demonstrated the fistulous tract and relation of the transverse colon and ascending colon (yellow arrow).

**Figure 2:**
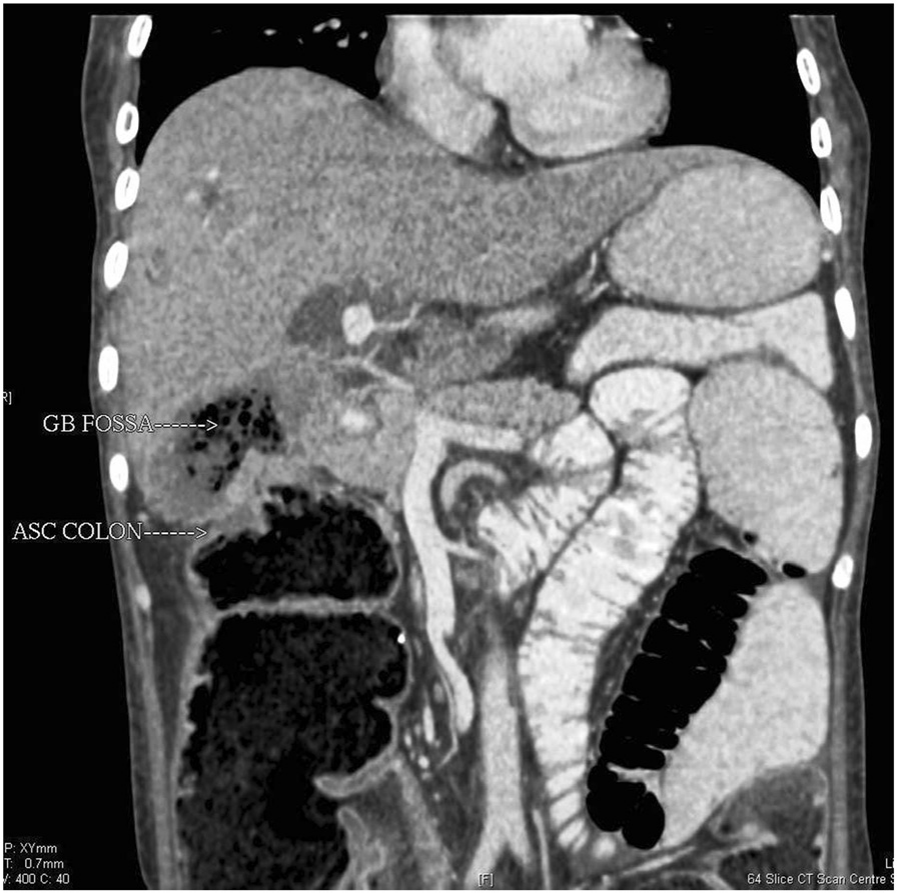
**Coronal reformatted image showed explicitly the hypoattenuating mass lesion with perilesional invasion and air loculi.** The narrow fistulous tract between the mass lesion and hepatic flexure was clearly shown with transmural invasion. The ascending colon was dilated.

**Figure 3:**
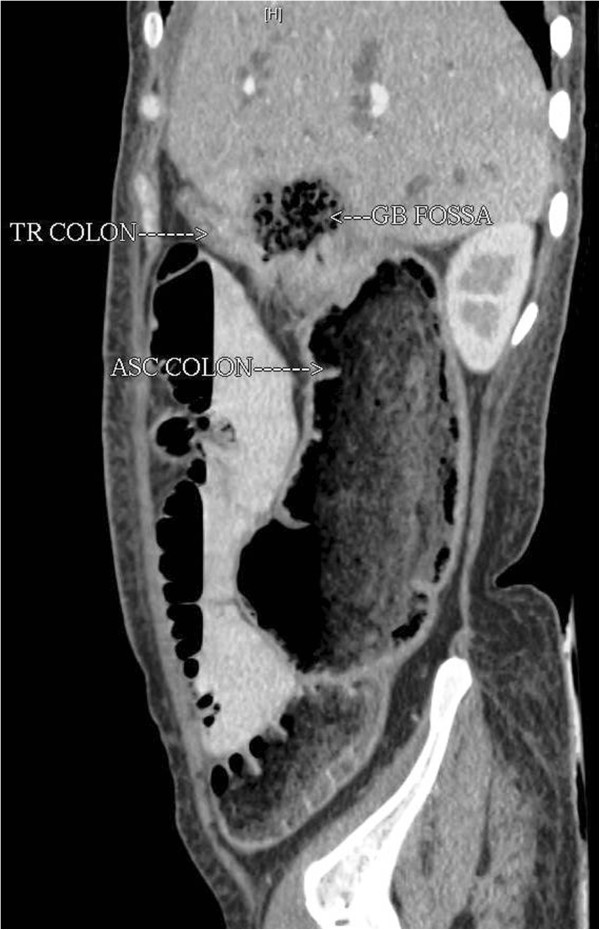
Sagittal reformatted image showed the pathology and anatomical location of the malignant cholecystocolic fistula.

Axial sections showedan ill-defined, hypoattenuating infiltrative soft tissue mass lesion in the gallbladder fossa. The lesion showed frank transmural invasion of the colonic wall with a discernible fistulous tract. Multiple air loculi were visible within the mass lesion. Contiguous axial sections demonstrated the fistulous tract and relation of the transverse colon and ascending colon (Figure 1A-C). The coronal reformatted image showed explicitly the hypoattenuating mass lesion with perilesional invasion and air loculi. The narrow fistulous tract between the mass lesion and hepatic flexure was clearly shown with transmural invasion. The ascending colon was dilated (Figure 2). The sagittal reformatted image showed the pathology and anatomical location of the malignant cholecystocolic fistula (Figure 3). The patient refused surgical treatment and was advised chemotherapy and radiotherapy.

## Discussion

Spontaneous enterobiliary fistulae are a complication of biliary disease or a disease of adjacent structures. They are usually associated with gallstones; however, peptic ulcer disease, abdominal trauma, Crohn’s disease, and malignancies of the biliary tract, bowel and head of pancreas, have also been implicated as causes [1-3]. The overall incidence of internal biliary fistula is 1.2 to 5.0% [4]. Cholecystoduodenal fistulae are the most frequent (75%), followed by cholecystocolic (10 to 20%), with a variety of other types being less common (15%) [3,5]. Cholecystocolic fistulae are rare in relation to GBC. Previous reports have showed images with subtle findings suggestive of cholecystocolic fistula. Prior to the advent of ultrasound and CT, contrast cholangiographic studies were used in the diagnosis of gallbladder and biliary tract diseases. In one study, 33.3% of cases showed involvement of hepatic flexure and mesocolon, as demonstrated by eccentric or circumferential wall thickening. In 2.3% of cases, a gallbladder mass lesion was seen closely abutting hepatic flexure with no obvious eccentric wall thickening [6].

GBC has been notorious to have a poor prognosis, with an overall 5-year survival rate reported to be 4% [7]. It is relatively rare in the West; however, a high incidence has been noted in various ethnic groups and populations [8]. It is a common occurrence in this geographic belt (eastern Uttar Pradesh, western Bihar and northern Madhya Pradesh provinces of North India), constituting 4.4% of all malignancies [9]. Studies from India suggest that cases from this geographic belt are more aggressive [10]. Clinical and radiological features of GBC overlap with those seen in cholelithiasis and cholecystitis, which can often causedelay in diagnosis until the disease is in the late stage. Correct preoperative diagnosis has improved considerably with the use of newer imaging techniques [11-13]. The most common CT finding in GBC, reported in various studies, is a mass that fills most of an enlarged and deformed gallbladder [7]. These masses are typically low in attenuation with variable enhancement. Polypoid masses are the second most common presentationand differentiation from benign polyps is based on size, with the polypoid masses typically larger than 1 cm. In a study of 69 patients with GBC who underwent exploratory laparotomy, 76.8% had liver involvement, 71% had lymph node involvement and 24.6% had peritoneal deposits [14]. Preoperative staging using multislice CT has an overall accuracy ranging from 83 to 86% [15,16].

## Conclusion

The endemic zone of North India comes across aggressive behavior of GBC. Imaging can be definitive and conclusive, and can obviate surgical exploration in appropriately selected cases. A combined approach using noninvasive diagnostic methods and percutaneous aspiration biopsies may reduce the number of explorative laparotomies in advanced cases of GBC. Multidetector scanners are a powerful modality to define the extent, anatomical information and associated complications of GBC.

## Consent

Written informed consent was obtained from the patient for publication of this Case Report and accompanying images. A copy of the written consent is available for review by the Editor-in-Chief of this journal.

## Abbreviations

CT: Computed tomography; FNAC: Fine needle aspiration cytology; GBC: Gallbladder carcinoma; MDCT: Multidetector computerized tomography.

## Competing interests

The authors declare that they have no competing interests.

## Authors’ contributions

ANDD: Conceived and designed the study. Drafted the manuscript and contributed to the intellectual content and given final approval of the version to be published. SK: Involved in data acquisition and data interpretation and has participated in design of study and intellectual content. SR: Has helped in data acquisition and image analysis and helped in drafting and revising the manuscript. BNM: Participated in data acquisition and data analysis and participated in its design. All authors read and approved the final manuscript.
